# Characterization and diagnostic value of the gut microbial composition in patients with minimal change disease

**DOI:** 10.3389/fphys.2022.1070569

**Published:** 2022-12-06

**Authors:** Yiding Zhang, Yukun Zhou, Wen Cui, Zhihui Wang, Xuemei Wang, Feng Wu, Peipei Wang, Ting Wang, Wei Yu, Li Wang, Jin Shang, Zhanzheng Zhao

**Affiliations:** ^1^ Department of Nephrology, The First Affiliated Hospital of Zhengzhou University, Zhengzhou, China; ^2^ School of Medicine, Zhengzhou University, Zhengzhou, Henan, China; ^3^ Department of Infectious Diseases, The First Affiliated Hospital of Zhengzhou University, Zhengzhou, China; ^4^ Biobank of The First Affiliated Hospital of Zhengzhou University, Zhengzhou, Henan, China; ^5^ Laboratory Animal Platform of Academy of Medical Sciences, Zhengzhou University, Zhengzhou, Henan, China; ^6^ Laboratory of Nephrology, The First Affiliated Hospital of Zhengzhou University, Zhengzhou, Henan, China

**Keywords:** minimal change disease, gut microbiota, non-invasive diagnosis, fecal microbiota transplantation, gut-kidney axis

## Abstract

**Background:** Minimal change disease (MCD) is one of the most common causes of primary nephrotic syndrome with high morbidity. This study aimed to explore the typical alterations of gut microbiota in MCD and establish a non-invasive classifier using key gut microbiome. We also aimed to evaluate the therapeutic efficiency of gut microbiota intervention in MCD through animal experiments.

**Methods:** A total of 222 stool samples were collected from MCD patients and healthy controls at the First Affiliated Hospital of Zhengzhou University and Shandong Provincial Hospital for 16S rRNA sequencing. Optimum operational taxonomic units (OTUs) were obtained for constructing a diagnostic model. MCD rat models were established using doxorubicin hydrochloride for exploring the therapeutic efficiency of gut microbial intervention through fecal microbiota transplantation (FMT).

**Results:** The α-diversity of gut microbiota decreased in MCD patients when compared with healthy controls. The relative abundance of bacterial species also changed significantly. We constructed a diagnostic model based on eight optimal OTUs and it achieved efficiency of 97.81% in discovery cohort. The high efficiency of diagnostic model was also validated in the patients with different disease states and cross-regional cohorts. The treatment partially recovered the gut microbial dysbiosis in patients with MCD. In animal experiments, likewise, the gut microbiota changed sharply in MCD rats. However, gut microbial interventions did not reduce urinary protein or pathological kidney damage.

**Conclusion:** Gut Microbiota shifts sharply in both patients and rats with MCD. Typical microbial changes can be used as biomarkers for MCD diagnosis. The gut microbiota compositions in patients with MCD tended to normalize after treatment. However, the intervention of gut microbiota seems to have no therapeutic effect on MCD.

## 1 Introduction

Minimal change disease (MCD), the disease which were prevalent in children and elderly people, is one of the most common causes of primary nephrotic syndrome in China (Ng et al., 2018). In Central China, MCD has accounted for approximately 37.86% of cases of primary nephrotic syndrome in patients who are under 20 years old in the past decades (Hu et al., 2020). The prevalence of MCD has also increased significantly in China in recent years (Xu et al., 2016; Yang et al., 2018). Severe edema and massive proteinuria are the most common clinical features of MCD, and some patients may become oliguric or even anuric signifying renal dysfunction. The contraindications, such as solitary kidney, severe hypertension, and hemorrhagic tendency may prevent patients from renal biopsy. However, there is a paucity of specific molecular markers for the MCD diagnosis, which brings challenges to the diagnosis and treatment of the disease.

The gut microbiota plays an indispensable role in maintaining the homeostasis of the human gut’s internal environment (Rooks and Garrett, 2016; Lin and Zhang, 2017). An increasing number of studies have revealed that the gut microbes can cause the occurrence and development of various diseases (Ma et al., 2019; Ren et al., 2020; Zhao et al., 2020; Zhang et al., 2021). Specific changes in the gut microbiome or related metabolites can contribute to the early diagnosis and differential diagnosis (Ren et al., 2019; Yu et al., 2020; Ren et al., 2021). Nevertheless, the relationship between gut microbiota and MCD remains unknown. Whether gut microbiota can become a noninvasive diagnostic tool or a therapeutic target of MCD is also unclear.

Therefore, the first aim of the present study was to explore and describe the alteration of the gut microbiota composition in patients with MCD. The second target was to establish a non-invasive diagnostic model for MCD. We also investigated the role of gut microbiota in the pathogenesis of MCD and investigated whether gut microbial interventions can alleviate or even treat MCD in animal experiments.

## 2 Materials and methods

### 2.1 Study population

**FIGURE 1:**
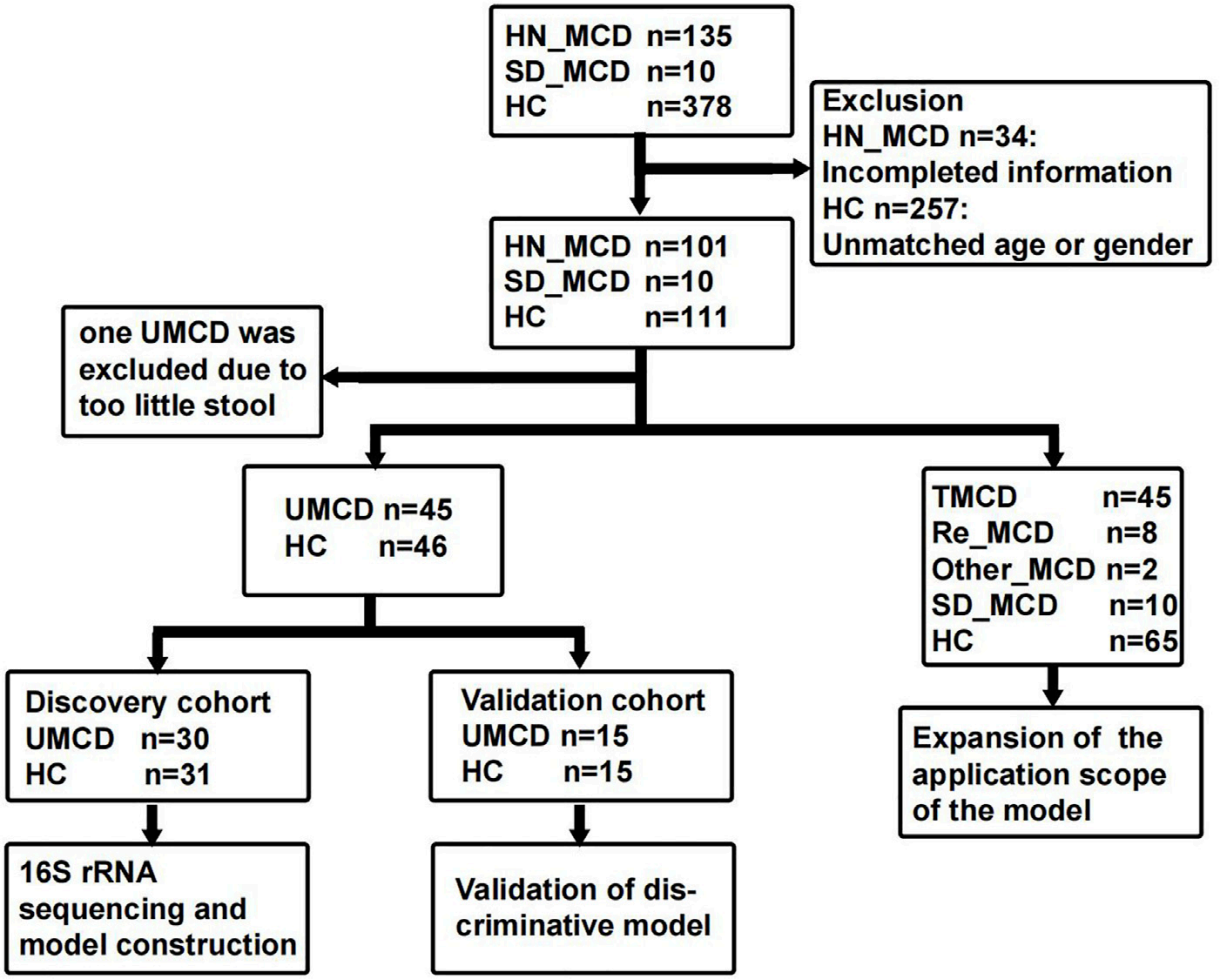
Study design and flow diagram. The fecal samples including MCD patients and healthy controls from two medicine centers of China were collected. After exclusion, a total of 222 fecal samples were enrolled in the study. In 111 fecal samples from patients with MCD, 101 were from the First Affiliated hospital of Zhengzhou University in Henan province and 10 were from Shandong Provincial Hospital. In 101 HN_MCD patients, 46 of them were first onset MCD without treatment and were defined as UMCD, while the rest was composed of 45 treated MCD, eight relapse MCD and two other types of MCD. Ten MCD patients from Jinan were also without a history of application of drugs. Except one sample with too little stool, 45 UMCDs and matched HCs were randomly divided into discovery cohort and validation cohort. We compared the gut microbial alteration in discovery cohort and selected key OTUs as biomarker to construct diagnostic model. Then we tested the efficiency in validation cohort. We also validated the diagnostic efficiency in SD_MCD, Re_MCD and TMCD cohorts to explore the applicable population and scope. MCD, minimal change disease; HN_MCD, MCD patients from Henan province; SD_MCD, MCD patients from Shandong province; HC, healthy controls; UMCD, untreated MCD; TMCD, treated MCD; Re_MCD, relapse MCD.

The study was designed based on the principle of prospective specimen collection and retrospective blinded evaluation (Pepe et al., 2008). A total of 135 MCD patients who were hospitalized in the First Affiliated Hospital of Zhengzhou University from May 2021 to February 2022, were included in this study. Each patient was pathologically diagnosed with MCD in the hospital. The exclusion criteria were as follows: 1) The presence of other metabolic diseases such as diabetes mellitus and hyperthyroidism. 2) Combining with other renal pathological lesions such as IgA nephropathy. 3) A history of antibiotic or probiotic therapy in the past three months. 4) The presence of other organ infections, such as pneumonia and gingivitis. After strictly applying inclusion and exclusion criteria, we recruited 101 MCD patients for further research, including 46 untreated MCD patients (UMCD) and 45 treated MCD patients (TMCD) who received treatment with corticosteroid or immunosuppressants. The median duration of treatment for TMCD patients was 407 (123, 918.5) days. In addition to these patients, there were eight patients who relapsed after complete remission and two patients obtained complete remission and just came for a recheck. Moreover, we collected another 10 fecal samples from first-diagnosed MCD patients in Shandong Provincial Hospital (JN_MCD) from July 2021 to February 2022. All healthy volunteers were recruited from the medical examination center of the First Affiliated Hospital of Zhengzhou University and had no history of antibiotic or probiotic treatment within 3 months prior to sample collection. Besides fecal samples, the demographic characteristics and laboratory indicators of each participant were also obtained. The flow chart is shown in Figure 1 and the baseline characteristics are shown in Supplementary Table S1.

### 2.2 Ethical approval

All participants included in this project signed informed consent in accordance with the Declaration of Helsinki. The First Affiliated Hospital of Zhengzhou University’s Ethics Review Committee granted ethical approval for the study and the ethics review approval ID was “2021-KY-838.” The samples collected from patients and healthy volunteers were stored in the Biobank of The First Affiliated Hospital of Zhengzhou University and the National Human Genetic Resources Sharing Service Platform (Grant No. 2005DKA21300).

### 2.3 16s rRNA sequencing

Fecal samples collected from all participants were temporarily stored in 4°C environment and then transferred to −80°C environment within 2 h for further analyses. DNA extraction from fecal samples was performed as previously described (Chen et al., 2011; Peng et al., 2018) using E.Z.N.A.^®^ Stool DNA Kit (Omega Bio-tek, Inc. GA). Shanghai MoBio Biomedical Technology Co., Ltd. provided technical support using the Miseq platform (Illumina Inc. United States) per the manufacturer’s protocols. The primers F1 and R2 (5’- CCTACGGGNGGCWGCAG -3’ and 5’-GACTACHVGGGTATCTAATCC-3’), which correspond to positions 341 to 805 in the *Escherichia coli* 16S rRNA gene, were used to amplify the V3-V4 region by PCR. PCR amplification of the V3-V4 region of the 16S rRNA gene and Illumina paired-end sequencing was performed according to a previous description. To obtain the clean data, we treated the Raw data using USEARCH (version 11.0.667) with the following criteria: 1) Sequences of each sample were extracted using each index with zero mismatch; 2) Sequences with overlap less than 16 bp were discarded; 3) Sequences less than 400 bp after merge were discarded; 4) Sequences with the error rate of the overlap greater than 0.1 were discarded. The quality-filtered sequences were clustered into unique sequences and sorted in order of decreasing abundance. According to UPARSE OTU analysis pipeline, the representative sequences were identified using UPARSE, and singletons were omitted in this step. Operational Taxonomic Units (OTUs) were obtained based on 97% similarity after chimeric sequences were removed using UPARSE (version 7.1 http://drive5.com/uparse/), and were annotated using the SILVA reference database (SSU138) (Edgar, 2013). The phylogenetic affiliation of the16S rRNA gene sequence was analyzed with a confidence threshold of 70% (Wang et al., 2007).

### 2.4 Microbial community analysis

Ace and Observed OTU indexes were calculated to compare the α-diversity in each group using Mothur v1.42.1. The non-parametric Mann-Whitney U test was used to test for statistical differences (R 3.6.0 package stats). A nonparametric Kruskal-Wallis test was used in the comparison of multiple groups. Principal Coordinate Analysis (PCoA) plots were generated by the QIIME (v 1.9.1) pipeline to visualize the weighted and unweighted UniFrac dissimilarity and the β-diversity (R. package vegan 2.5-7). Non-metric multidimensional scaling (NMDS) was used to visualize the composition differences between different groups. Analysis of similarity (ANOSIM) was used to compare the between-group and within-group components differences in different groups. Diagrams of average relative abundance compositions were used to describe the alteration of gut microbiota. The heatmap was used to visualize the discrepancy of relative abundance in key OTUs. The associations between key OTUs and clinical library laboratory results were explored and shown *via* the Strong, Prosperous, and Resilient Communities Challenge (SPARCC). PICRUSt2 v2.4.1 (https://github.com/picrust/picrust2/wiki) was used to predict the profile of functional abundances in gut microbiome.

### 2.5 Construction and validation of discriminative model

In constructing the identification model, we used the propensity scoring method to match UMCDs and healthy controls (HCs) in a 1:1 ratio according to gender and age. The matched cohort was subsequently divided into a discovery cohort and a validation cohort. After comparing the alteration of gut microbiota and profiles of OTUs in the discovery cohort, we used the Wilcoxon rank sum test to determine the significant OTUs (*p* < 0.05) for further analysis (Shen et al., 2022). All the key OTUs needed to meet the following conditions: 1) The abundance was greater than 0.5% in at least one sample. 2) the *p*-value of Wilcoxon rank sum test was less than 0.01. 3) Mean decrease in accuracy value in random forest model was more than 0.0001. We also tested the significant differences using the R 3.6.0 Package “ALDEx2”. The candidate OTUs obtained from the discovery cohort were used for the five-fold cross-validation. The verification was performed on a random forest model for feature critical OTUs (importance value > 0.001) as biomarkers for predicting the possibility of disease (POD) values of each individual. The receiver operating characteristic curve (ROC) was drawn (R 3.3.0, pROC package) to evaluate the constructed model, and the area under the curve (AUC) was used to represent the discrimination capability of the model as described in a previous study (Qiu et al., 2016). The related scripts of the model were stored at https://github.com/Neal050617/RFCV. The text cohort was used to validate the diagnostic effectiveness. We also tried to verify the effectiveness of the model in external cohorts to observe the application scope of the model. The statistical analyses were performed using R 3.6.0 (http://www.R-project.org/) and SPSS 24.0 software. Graphpad prism v 8.0 was used to draw the dot and bar charts.

### 2.6 Establishment of the minimal change disease rat model

The methods were performed in accordance with relevant guidelines and regulations and approved by the Ethics Review Committee of the First Affiliated Hospital of Zhengzhou University. Healthy and clean male Sprague-Dawley (SD) rats weighting 70–80 g were obtained from Spaefer Biotechnology Co., Ltd. All rats were fed in the breeding area of experimental animal center of Zhengzhou University under similar conditions with a 12 h light/dark cycle at 22°C–24°C after birth. The rats were transferred to the cages in experimental area at 3–4 weeks old and provided a standard diet and drinking water *ad libitum*. There were seven rats in a group (three cages with two rats and one cage with one rat). The cages were connected with a ventilation system and the sterilized cage. Dry bedding was replaced every two days. After 2 weeks of adaptive feeding, doxorubicin hydrochloride (DOX, Shanghai Macklin Co., Ltd.) was used to establish the rat model (MCD rats, *n* = 7) *via* tail-vein injection at a dose of 4 mg/kg in week 0 and 3.5 mg/kg in week 1, learning and improving from the Bertani method (Bertani et al., 1982). The controls were rats injected with the same dose of normal saline (Con rats, *n* = 7).

### 2.7 Fecal microbiota transplantation treatment for minimal change disease rats

Fecal microbiota transplantation (FMT) has been widely utilized in researches involving animal models (Daniel et al., 2022; Zhong et al., 2022). We also performed FMT on MCD rat model to explore the therapeutic or aggravative effects of gut microbiota. We collected fecal samples from Con and MCD rats and pooled them according different groups. The fecal samples were weighted and diluted with normal saline to make the concentration of fecal suspension about 120 mg/ml. After removing large particles with filter screen, the glycerin was added to about 20% concentration. Then the stool suspension was stored at −80°C environment until FMT. Before FMT, an antimicrobial solution made up of Ampicillin 0.1 g/L, Vancomycin 0.5 g/L, Neomycin 1 g/L, and Metronidazole 1 g/L replaced distilled water for daily water intake. As a substitute for drinking water, the antimicrobial solution was changed every two days and the process of gut microbiota cleaning continued for 2 weeks. We collected feces from control and MCD rats every week to prepare the fecal bacterial solution. Fresh fecal samples were diluted with normal saline at a concentration of about 120 mg/ml. Then, the bacterial solution was filtered to remove large particles, after which glycerol was added to adjust the concentration of glycerol in solution to 20%, and stored at −80°C.

After tail-vein injections with doxorubicin hydrochloride, 35 more SD rats from the same batch as before were fed the antimicrobial solution, as described previously, for 2 weeks. Two groups with a total of 14 rats had gut microbiome cleaning before (MC + MCD group, *n* = 7) and after (MCD + MC group, *n* = 7) doxorubicin hydrochloride injection respectively to eliminate the influence of the application order of antibiotic solution on modeling. The remaining 21 rats, separated into three groups, received gavage with normal saline (MCD + NS group, *n* = 7) or fecal bacterial solution collected from control rats (MCD + FC group, *n* = 7) or MCD rats (MCD + FM group, *n* = 7). Gavage with 1 ml of the bacterial solution was performed every 2 days for 5 weeks.

### 2.8 End point and evaluation of rat model

Feces and urine samples were collected once a week and transferred to a −80°C environment. Urinary protein was assessed to estimate the severity of MCD. The fecal samples underwent 16s rRNA analyses to compare the microbial compositions in different groups. At the end of the eighth week, we sacrificed all rats and collected the plasma and kidney samples. After centrifugation at 4°C and 4,000 rpm for 10 min, the plasma samples were immediately stored at −80°C. Kidney specimens were prepared for further pathological observation under light and electron microscopes. HE (hematoxylin-eosin) staining was used to suggest the general pathological injury and local inflammation, PAS (periodic Acid-Schiff) staining was used to observe the changes of glomerular basement membrane thickness, and Masson staining was used to show fibrosis and inflammatory changes in tissues.

## 3 Results

### 3.1 Microbial alteration in UMCD patients

**FIGURE 2:**
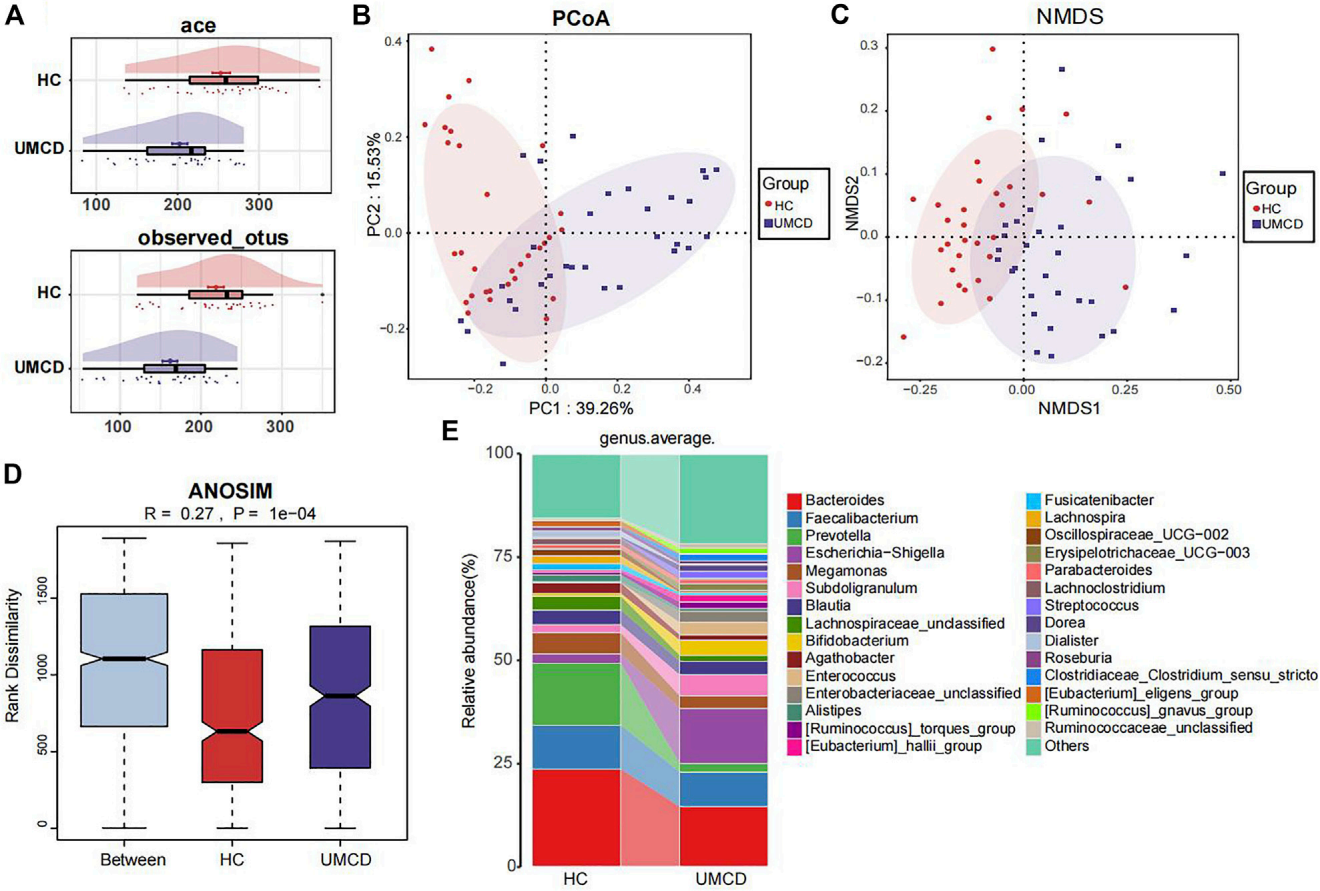
Comparison of gut microbiota composition in discovery cohort (UMCD = 30, HC = 31). **(A)** The ace index (*p* < 0.001) and observed OTUs (*p* < 0.001) showed α-diversity decreased in UMCD. **(B)** PCoA diagram showed the weighted UniFrac distance had obviously difference between UMCD and HCs. **(C)** NMDS showed different distances in composition of gut microbiota. **(D)** ANOSIM diagram showed significant differences between HCs and UMCDs. **(E)** Average relative abundance at genus level in HCs and UMCDs were compared. PCoA, principal coordinate analysis; NMDs, Non-metric multidimensional scaling; ANOSIM, analysis of similarity.

UMCD patients and matched HCs were randomly divided into a discovery cohort and a validation cohort in a 2:1 ratio (Baseline features are shown in Supplementary Table S2). In the discovery cohort, we compared the gut microbial composition and found that it changed significantly in UMCDs. The Ace index (*p* < 0.001) and observed OTUs (*p* < 0.001) were decreased in UMCDs, representing the reduction of the α-diversity (Figure 2A). The PCoA diagram shows that the weighted UniFrac distance also changed significantly in PC1 and PC2 (Figure 2B). The NMDS analysis also revealed a significant discrepancy between the two groups (Figure 2C). The diagram of ANOSIM shows that the component differences in HC and UMCD groups were significant (Figure 2D). Moreover, we compared the average abundance in the genus level between two groups. *Prevotella*, which is commonly considered beneficial to human health because of its contributions in weight control and improving glucose metabolism (Kovatcheva-Datchary et al., 2015; Christensen et al., 2019), was found to decrease sharply in our study (*p* = 0.042). On the contrary, an increase in harmful microbiomes such as *Escherichia−Shigella* was found in UMCDs (Figure 2E, *p* = 0.0106). The alteration of relative abundance in phylum and genes levels was shown in Supplementary Tables S3, S4, respectively. These results indicated that the gut microbial composition changed significantly in UMCDs, as there was a decrease in beneficial bacteria and an increase of harmful bacteria.

### 3.2 Construction and validation of the discriminative model

**FIGURE 3:**
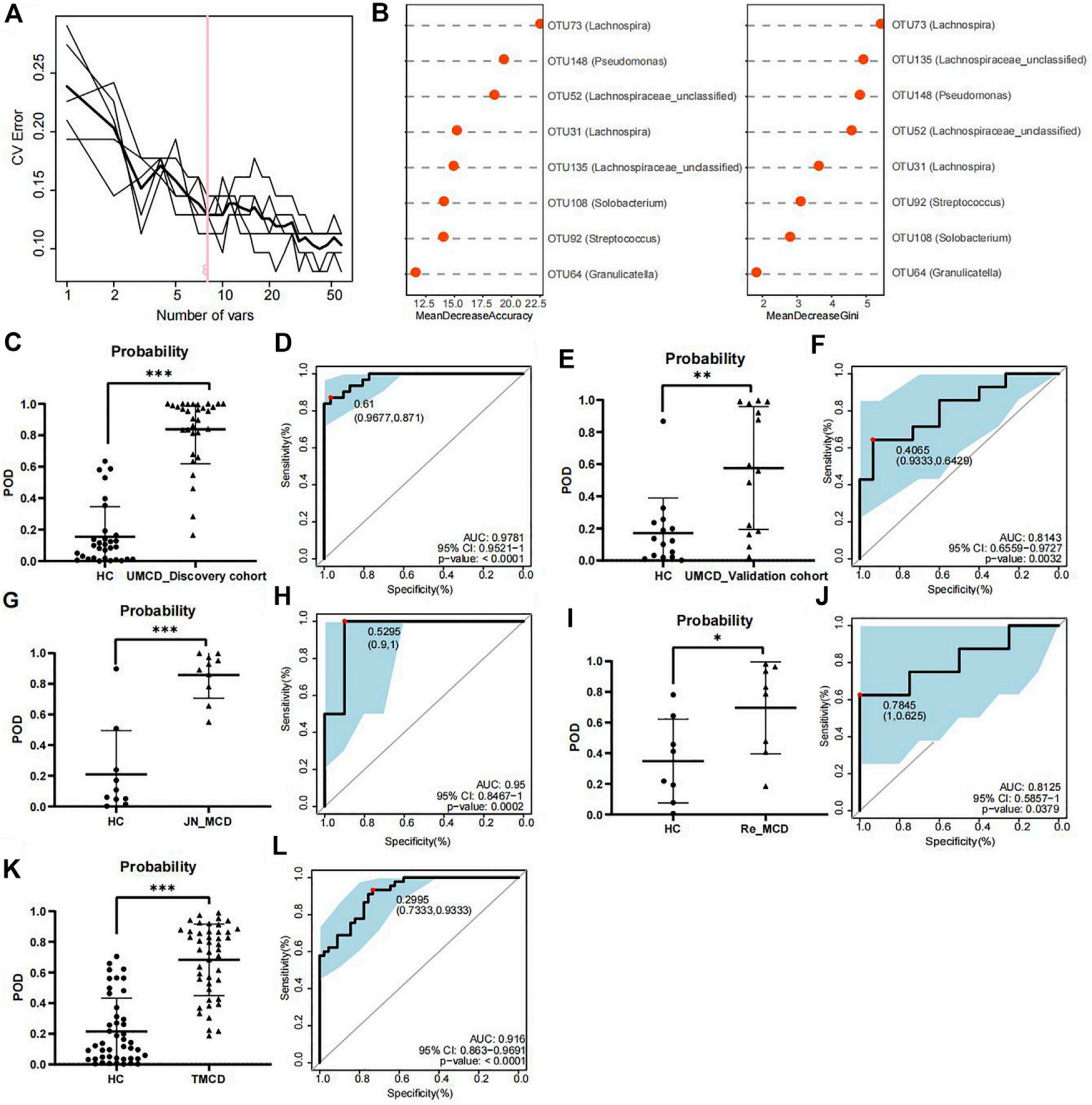
Construction and validation of discriminative model. **(A)** Random forest model showed when containing eight OTUs, the model had lower CV error with fewer variables. **(B)** Mean decrease accuracy and mean decrease gini showed the contribution values of eight selected OTUs. **(C)** Probability of disease was obvious higher in UMCDs (*n* = 30) than that in HCs (*n* = 31) in discovery cohort. **(D)** Discriminative model based on obtained microbial biomarkers showed good diagnostic efficiency with an AUC of 0.9781 in discovery cohort. **(E)** Probability of disease was obvious higher in UMCDs (*n* = 15) than that in HCs (*n* = 15) in validation cohort. **(F)** Discriminative model showed good diagnostic efficiency with an AUC of 0.8143 in validation cohort. **(G)** Probability of disease was obvious higher in JN_MCDs (*n* = 10) than that in HCs (*n* = 10). **(H)** Discriminative model showed good diagnostic efficiency with an AUC of 0.95 in JN_MCDs and HCs. **(I)** Probability of disease was obvious higher in Re_MCDs (*n* = 8) than that in HCs (*n* = 8). **(J)** Discriminative model showed good diagnostic efficiency with an AUC of 0.8125 in Re_MCDs and HCs. **(K)** Probability of disease was obvious higher in TMCDs (*n* = 45) than that in HCs (*n* = 45). **(L)** Discriminative model showed good diagnostic efficiency with an AUC of 0.916 in TMCDs and HCs. CV, coefficient of variation; OTU, operational taxonomic unit POD, possibility of disease; ROC, receiving operational curve; AUC, area under curve. *, *p* < 0.05; **, *p* < 0.01; ***, *p* < 0.001.

To explore whether the gut flora could be used as biomarkers for MCD diagnosis, we performed five-fold cross-validation on a random forest model to gain optimum OTUs. We found that when it contained eight OTUs, the model had a lower CV error with fewer variables (Figure 3A). We obtained OTU64 (*Granulicatella*), OTU92 (*Streptococcus*), OTU108 (Solobacterium), OTU135 (Lachnospiraceae*_unclassified*), OTU31 (*Lachnospira*), OTU52 (Lachnospiraceae*_unclassified*), OTU148 (*Pseudomonas*), and OTU73 (*Lachnospira*) for model construction (Figure 3B). The comparison of average abundance of eight OTUs between UMCDs and HCs in discovery cohort was shown in Supplementary Table S5 and Supplementary Figure S1. The validation results of the obtained OTUs using ALDEx2 are shown in Supplementary Table S6. The classifier showed that UMCDs in the discovery cohort had a higher POD value level (Figure 3C) and they had a high efficiency with an AUC of 97.81% (cut-off value: 0.61, sensitivity: 0.871, specificity: 0.9677, Figure 3D). In the validation cohort, the classification accuracy was also high, with an AUC of 81.43% (cut-off value: 0.4065, sensitivity: 0.6429, specificity: 0.9333), suggesting a good diagnostic ability to the model (Figures 3E,F). The comparison of the key OTUs in validation cohort was shown in Supplementary Table S7.

In the external validation cohort composed of 10 cases of first-onset MCD from Jinan and 10 matched HCs (Baseline characteristic shown in Supplementary Table S8, comparison of eight OTUs shown in Supplementary Table S9). The classifier showed good discriminative efficiency as well, with an AUC of 95% (cut-off value: 0.5295, sensitivity: 1, specificity:0.9, Figures 3G,H), proving that the diagnostic model had applicability in different regions.

To verify whether the classifier is applicable in other types of MCD patients, we further validated the model in patients with MCD relapse (the Re_MCD cohort, baseline characteristics are shown in Supplementary Table S10, average abundance of eight OTUs is shown in Supplementary Table S11) and treated MCD patients (the TMCD cohort, baseline characteristics are shown in Supplementary Table S12, average abundance of eight OTUs is shown in Supplementary Table S13). Interestingly, the diagnostic model showed high classification precision with an AUC of 81.25% in the Re_MCD cohort (cut-off value: 0.7845, sensitivity: 0.625, specificity: 1, Figures 3I,J) and 91.6% in the TMCD cohort (cut-off value: 0.2995, sensitivity: 0.9333, specificity: 0.7333, Figures 3K,L).

The results of the development and validation of the model revealed that our diagnostic model based on crucial OTUs had high accuracy and was suitable for MCD patients in different regions and different disease states.

### 3.3 Comparison of the microbial community in UMCDs, TMCDs, and HCs

**FIGURE 4:**
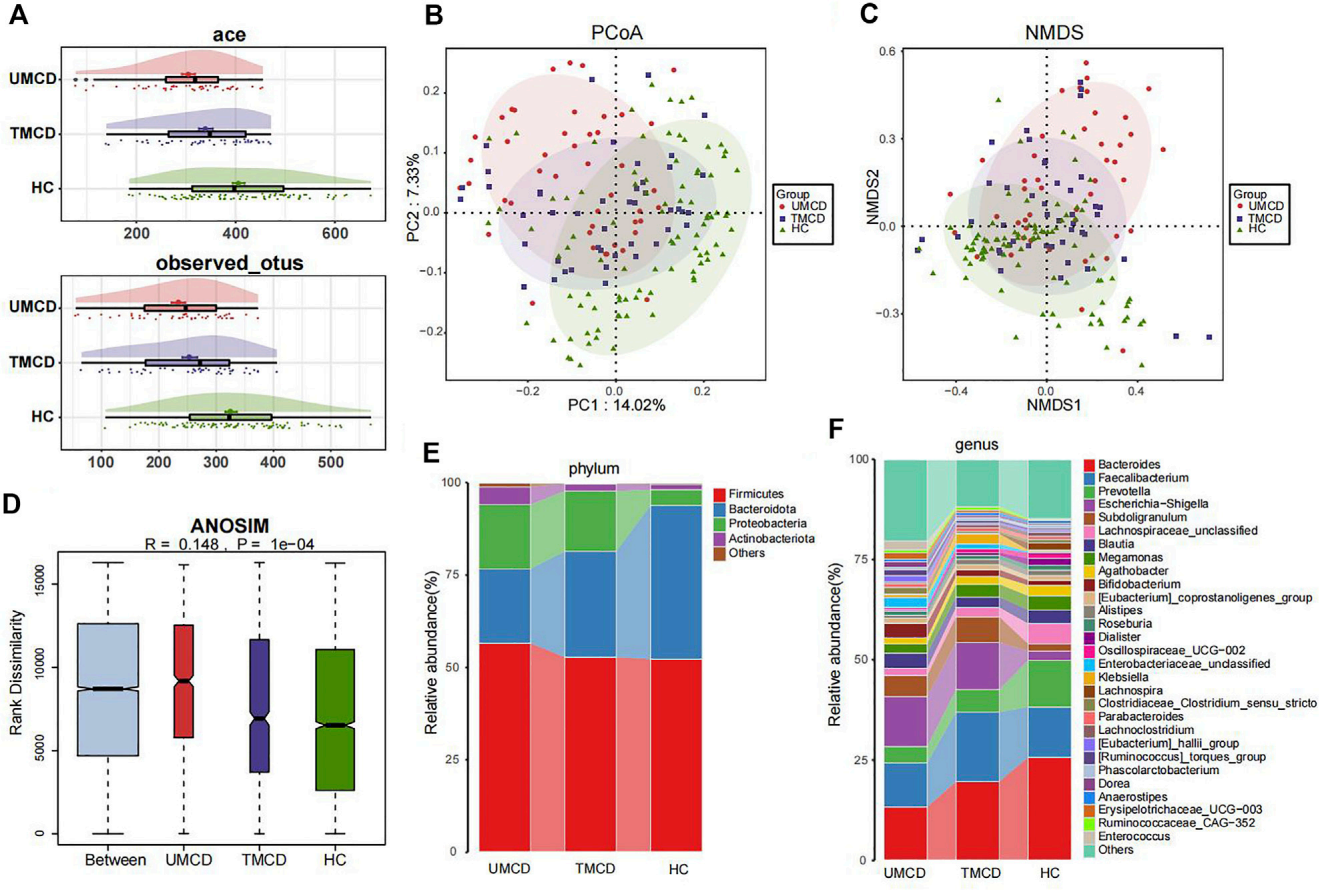
Comparison of gut microbiota compositions among UMCD (*n* = 45), TMCD (*n* = 45) and HC groups (*n* = 91). **(A)** The ace indexes and observed OTUs had no difference in UMCDs and TMCDs, and both of them were lower than those in HCs (Ace index: UMCD vs. HC, *p* < 0.001, TMCD vs. HC, *p* < 0.01; observed OTUs, UMCD vs. HC, *p* < 0.001, TMCD vs. HC, *p* < 0.001). **(B)** PCoA diagram showed that the weighted UniFrac distance got similar to HCs after treatment in patients with MCD. **(C)** NMDs diagram showed the compositions of gut microbiota were similar to HCs after treatment. **(D)** ANOSIM diagram showed significant difference among three groups. Average relative abundance at phylum **(E)** and genus **(F)** levels among three groups were compared.

Previous studies have shown that drugs, such as corticosteroids, tacrolimus, or metformin, can have varying degrees of impact on the host’s gut microbiota (Jiang et al., 2018; Pastor-Villaescusa et al., 2021; Petrullo et al., 2022). However, in our project, drug application did not seem to affect the effectiveness of the diagnostic model. We compared the gut microbial compositions in UMCD, TMCD and HC groups. The comparison of eight key OTUs in the established model was shown in Supplementary Figure S2. There was no statistically significant difference in the ace index and observed OTUs of α-diversity between the UMCD and TMCD groups (ace, *p* = 0.0718; observed OTUs, *p* = 0.1830). However, both indexes were significantly lower when compared to those in HCs (ace, UMCD vs. HC, *p* < 0.001; observed OTUs, UMCD vs. HC, *p* < 0.001; ace, TMCD vs. HC, *p* = 0.035; observed OTUs, TMCD vs. HC, *p* < 0.001; Figure 4A). The PCoA diagram shows unweighted UniFrac dissimilarity among the three groups (Figure 4B). The NMDS analysis also showed the distance differences of gut microbiota compositions of the three groups (Figure 4C). However, both PCoA and NMDS diagrams indicated the gut microbial composition of TMCD is the intermediate process of the transformation from gut microbiota profile in UMCD to that in HC. ANOSIM suggested the between-group component difference was greater than the within-group component difference (Figure 4D).

Then we compared the relative abundance of eight OTUs included in the diagnostic model. We found six OTUs of the eight showed partial recovery (Supplementary Table S14). The relative abundance of many bacteria also changed in a ladder-like manner, such as *Bacteroidota* at the phylum level (Figure 4E, Supplementary Table S15), *Bacteroides* and *Prevotella* at the genus level (Figure 4F, Supplementary Table S16). The increase of *Firmicutes*/*Bacteroidetes* ratio was thought to be related to many disease such as obesity, type 2 diabetes, and chronic kidney disease (Grigor’eva, 2020; Guan et al., 2020). In our research, the treatment also decreased the *Firmicutes*/*Bacteroidetes* ratio in MCD patients (Figure 4E). The results implied that the gut microbiota composition dynamically and partly recovered after treatment.

### 3.4 Correlation analysis between clinical indicators and crucial OTUs

**FIGURE 5:**
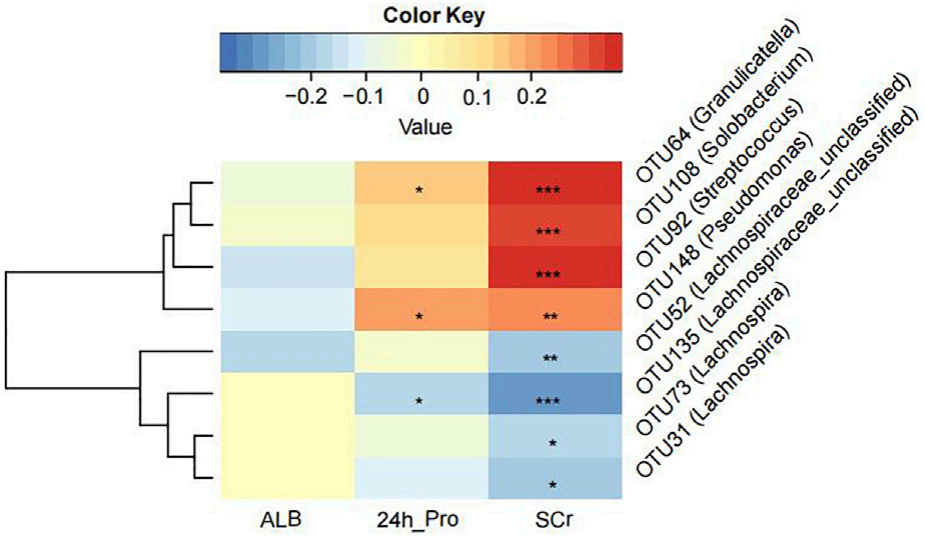
The Strong, Prosperous, and Resilient Communities Challenge (SPARCC) analysis was used to analyze the degree of correlation between key OTUs used for model construction and clinical indicators. ALB, albumin; 24 h_Pro, 24 h urine protein; SCr, serum creatinine. *, *p* < 0.05; **, *p* < 0.01; ***, *p* < 0.001.

We tried to explore the relationship between crucial OTUs and clinical indicators in all 100 MCD patients. We used SPARCC analysis to explore the relationship between the eight OTUs obtained from model construction and the results of laboratory examinations including 24-h urine protein, albumin and serum creatinine. Interestingly, the results proved that OTU108 (*Solobacterium*), OTU92 (*Streptococcus*), OTU148 (*Pseudomonas*) and OTU64 (*Granulicatella*) were all positively correlated with serum creatinine, which might affect the progression of MCD (Figure 5 and Supplementary Table S17). The other four OTUs all belonged to Lachnospiraceae, some of which were negatively correlated with serum creatine. OTU148 (*Pseudomonas*) and OTU64 (*Granulicatella*) were also positively correlated with 24-h urine protein level and OTU135 (Lachnospiraceae) was negatively correlated with 24-h urine protein level.

### 3.5 Clinical feature and microbial alteration in minimal change disease rats

**FIGURE 6:**
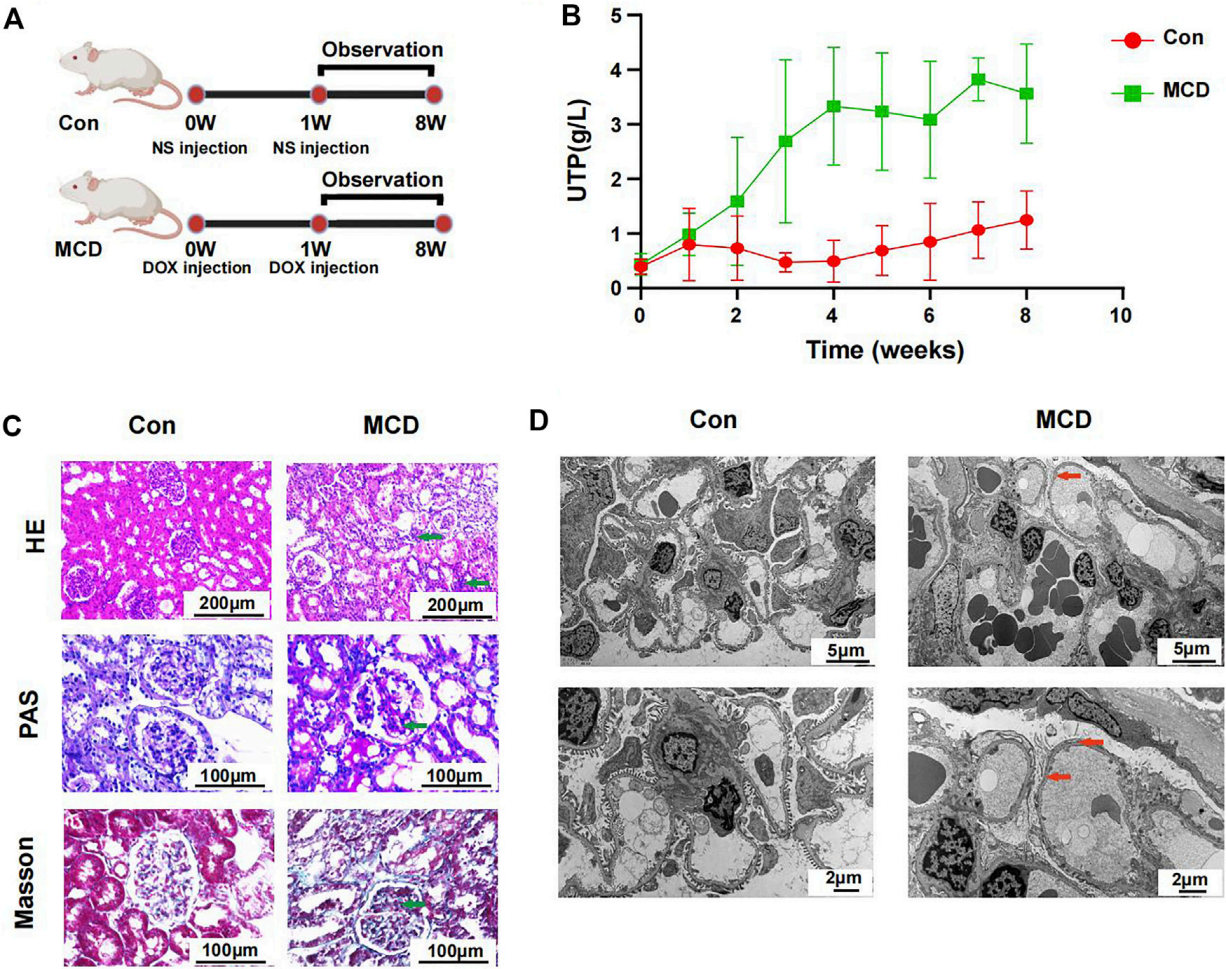
Modeling process and changes of urinary protein in Con group (*n* = 7) and MCD group (*n* = 7). **(A)** DOX injected *via* tail-vein at a dose of 4 mg/kg body weight in week 0 and 3.5 mg/kg body weight in week one in MCD group. The Con group was given the same dose NS. **(B)** Line chart showed the alteration of urinary protein after modeling. **(C)** HE, PAS and Masson staining of glomerulus in different groups. **(D)** Electron microscopy showed detailed pathological injury in each group. DOX, doxorubicin hydrochloride; NS, normal saline. HE, hematoxylin-eosin staining; PAS, periodic acid-schiff staining.

The modeling process and end points are shown in Figure 6A. As expected, rats in the MCD group had significantly more proteinuria from week three and it continued until week 8 (Figure 6B, Supplementary Figures S3A,B).

Pathological changes proved that MCD rat model were constructed successfully. Under the light microscope, we observed inflammatory reaction and fibrosis of the glomerulus through HE and Masson staining. The basement membrane showed thinner through PAS staining. We also observed necrosis of some renal tubules through different stains (Figure 6C). Under the electron microscope, we observed typical MCD changes, including extensive foot process fusion (Figure 6D).

**FIGURE 7:**
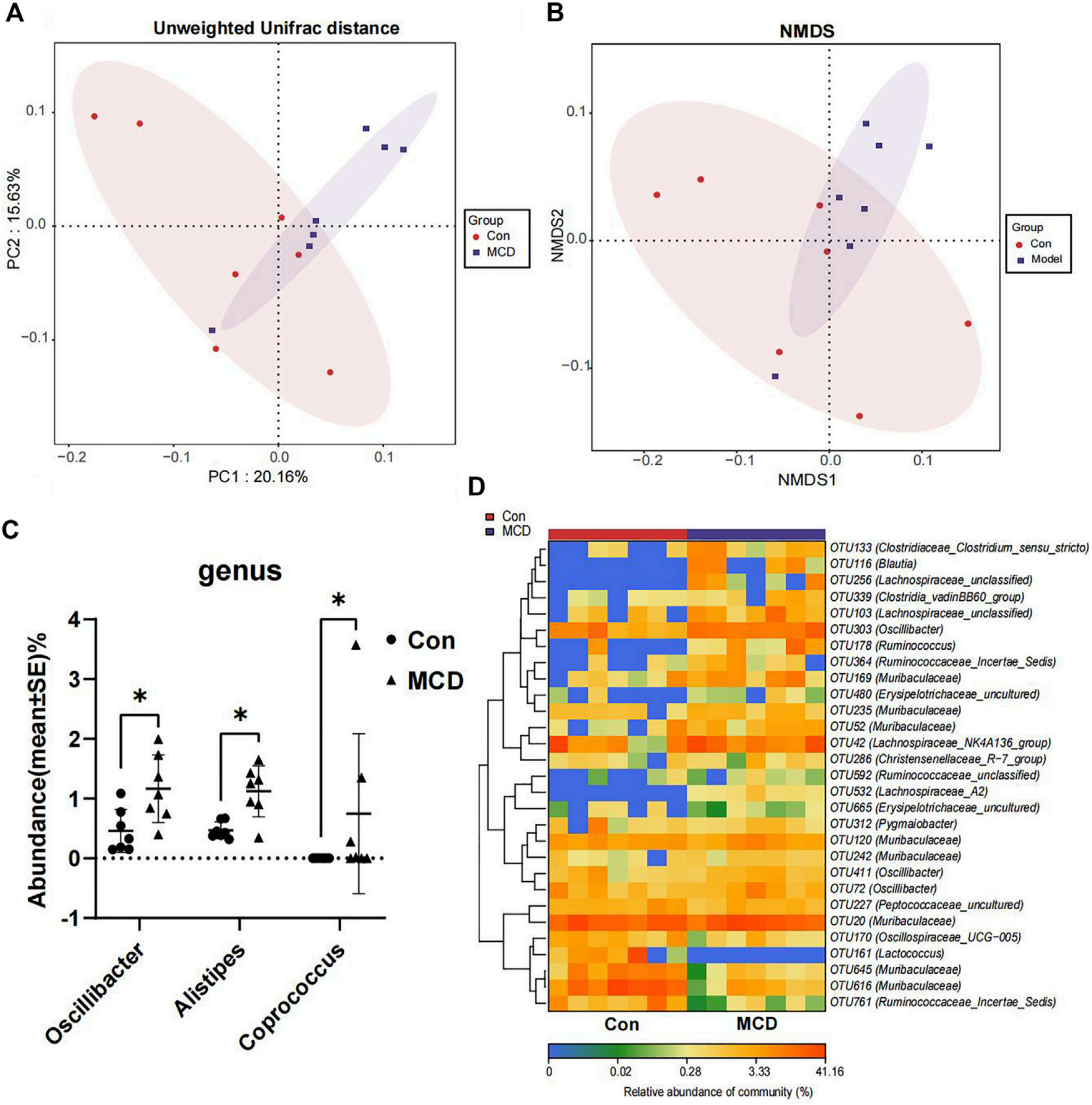
Comparison of gut microbiota composition of rats in Con group (*n* = 7) and MCD group (*n* = 7). **(A)** PCoA diagram showed the weighted UniFrac distance had obviously difference between Con group and MCD group. **(B)** NMDS diagram showed the difference in composition of gut microbiota. **(C)** There genera of bacteria changed markedly in MCD group. **(D)** Heatmap showed difference in relative abundance of key OTUs.

In week 4, rats in the MCD group developed heavy sustained proteinuria. We analyzed gut microbial compositions of fecal samples collected in week four by the 16S rRNA sequencing. These analyses suggested that microbiota compositions in MCD rats had a conspicuous alteration compared to the control group. PCoA diagrams showed that the Unweighted UniFrac distance had shape differences in PC1 and PC2 (Figure 7A). NMDS analyses also suggested significant alterations of the gut microbiome composition in rats with MCD (Figure 7B). According to the results of Wilcoxon’s rank-sumtest and ALDEx2, three genera (*Oscillibacter*, *Alistipes* and *Coprococcu*) had shape changes (Figure 7C). Heatmap of key OTUs also demonstrated that MCD rats had gut microbial alterations (Figure 7D, Supplementary Table S18). The results of animal experiments once again verified that MCD could lead to the alteration of gut microbiota.

### 3.6 Influence of the fecal microbiota transplantation intervention on rats with minimal change disease

Before the FMT intervention, we treated the rats with an antibiotic solution. To confirm whether the antibiotic solution had any effect, rats in the MC + MCD and MCD + MC groups underwent gut microbiota cleaning before and after modeling, respectively (Supplementary Figure S4A). There was no significant difference in the urinary protein levels in MC + MCD and MCD + MC groups after week 5 (Supplementary Figures S4B,C). The pathological lesions of the two groups were also similar (Supplementary Figures S5A,B), suggesting that the sequence of gut microbial clearing and modeling did not affect the phenotypes of MCD model.

**FIGURE 8:**
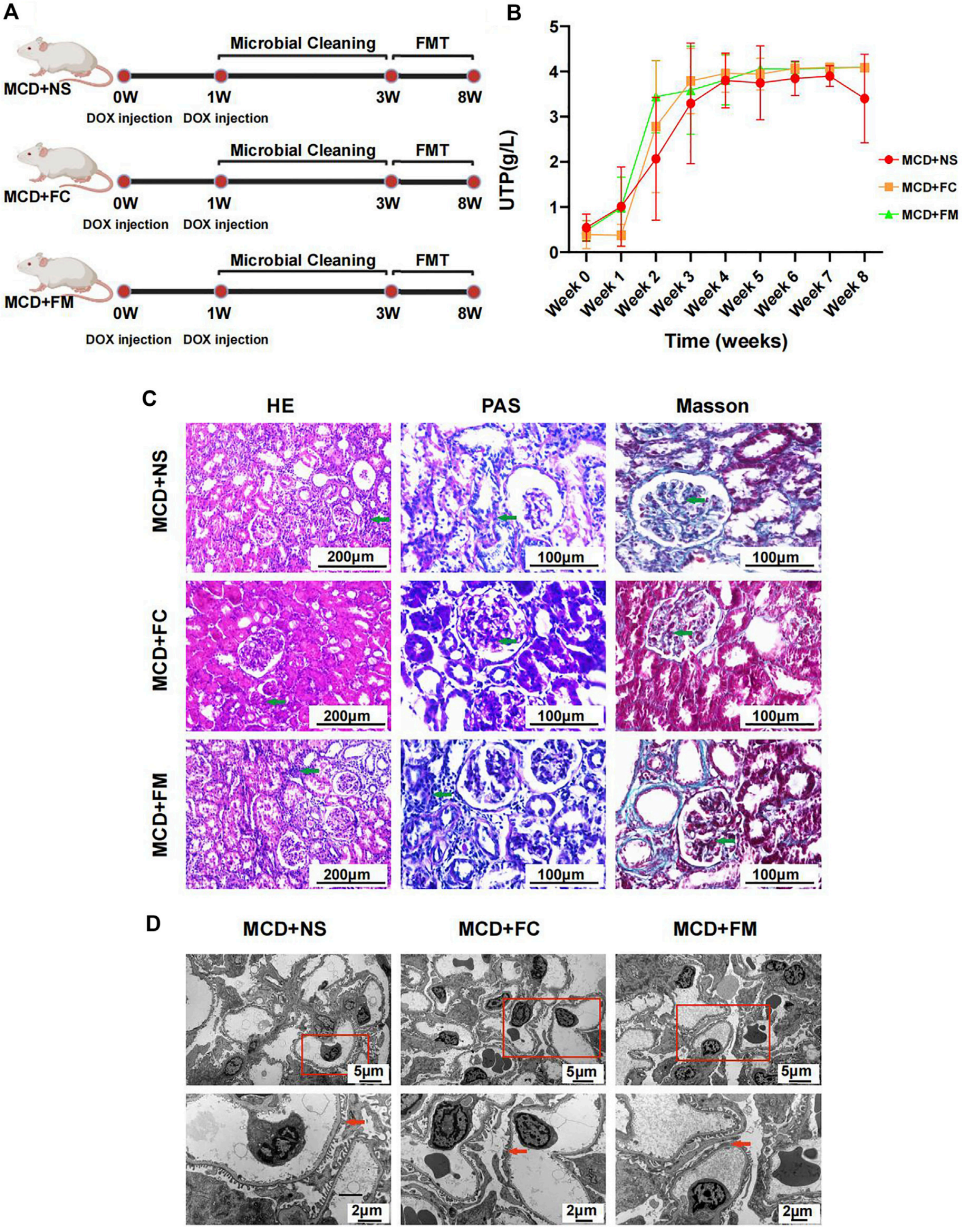
Modeling process and Pathological injury in MCD + NS group (*n* = 7), MCD + FC group (*n* = 7) and MCD + FM group (*n* = 7). **(A)** After modeling, rats in three groups were given a two-week gut microbiota cleaning. Then FMT continued from week three to week eight in three groups. **(B)** Line chart showed the alteration of urinary protein after modeling. **(C)** HE, PAS and Masson staining of glomerulus in different groups. **(D)** Electron microscopy showed detailed pathological injury in each group. FMT, fecal microbiota transplantation; FC, fecal microbiota transplantation with feces from Con group; FM, fecal microbiota transplantation with feces from MCD group.

Rats that underwent FMT with feces from the control group or the MCD group did not display any significant improvement in their disease. The modeling process is shown in Figure 8A. The urinary protein did not differ significantly among the three groups during the whole experiment period (Figure 8B, Supplementary Figures S6A,B). After sacrificing the rats in week 8, pathological renal injuries had no evident diversities when observed under light or electron microscopes. The infiltration of inflammatory cells and the aggravation of interstitial fibrosis were observed in the three groups of rats through HE and Masson staining (Figure 8C). Under electric microscope, the foot processes of podocytes in all three groups showed partial fusion (Figure 8D).

The difference in the gut microbiome composition between the MCD + FC group and the MCD + FM group was similar to that between the control group and the MCD group, suggesting successful transplantation. The PCoA diagrams showed different compositions in the Weight UniFrac distance (Supplementary Figure S7A). The between-group component difference was greater than the within-group component difference (Supplementary Figure S7B). Similar to the control group vs. the MCD group, *Oscillibacter* and *Alistipes* were also observed to be richer in the MCD + FM group than that in the MCD + MC group (Supplementary Figure S7C). The heatmap also shows a difference in the pattern of key OTUs between the two groups (Supplementary Figure S7D; Supplementary Table S19). These results suggested successful FMT in the project and showed that there was no significant effect of FMT on MCD.

## 4 Discussion

In this project, we collected and analyzed a total of 222 fecal samples from MCD patients and healthy volunteers in two medical centers in China. As far as we know, this is the first study to compare the composition of gut microbiota between patients with MCD and healthy controls. Typical microbial changes were observed in MCD patients. Unique gut microbiome profile could be used as biomarkers for MCD identification. The constructed discriminative model suggested good capability to distinguish MCD from HC, and the distinguishing model was also suitable to differentiate treated or relapse MCD cases. In subgroup analysis, we found that gut microbiota compositions in patients with MCD had a tendency to transform into those seen in healthy individuals when receiving medical treatment. Through animal experiments, we also discovered the alteration of gut microbiota in rats with MCD. We performed FMT interventions on MCD rats but found that it could not help improving the clinical symptoms or reduce pathological renal damage of MCD.

The gut microbiota composition was found to be altered in adult patients with idiopathic nephrotic syndrome (He et al., 2021). However, in children with primary nephrotic syndrome, the richness and diversity of gut microbiota were similar before and after 4-week-long initial therapy (Kang et al., 2019). Prior to our project, there was still a paucity of description of gut microbial profile for MCD pathological type. Before receiving medication, the gut microbial composition had already altered. The treatment seems to rectify the gut dysbiosis in patients with MCD. However, our diagnostic model still showed a good accuracy in TMCDs. We think it is because that the typical change remains stable even after starting medical treatment for patients with MCD. The results need to be confirmed in more studies, though. Although the mechanism of gut microbiota and renal injury in MCD patients is still unclear, the relationship between the two is quite close. In our study, the abundance of many short chain fatty acid (SCFA) producing bacteria, such as *Faecalibacterium* was reduced (Zhou et al., 2018). The decreased abundance of SCFAs, which plays an important role in the integrity of gut barrier (Liang et al., 2022), might destroy the gut barrier and make the inflammatory mediator enter the circulatory system. At the same time, decreased abundance and death of gut microbiome would produce a large amount of endotoxin, such as lipopolysaccharide (LPS) and muramyl dipeptide (MDP), leading activation of immune system and aggravation of renal injury. We inferred that it is one of the important ways for gut microbiota to aggravate kidney damage in MCD patients. Interestingly, *Bacteroides*, members of which were uremic toxin producers, was found decreased in patients with MCD. However, the similar results was observed in patients with membranous nephropathy (Shang et al., 2022). In addition, the increase of *Firmicutes*/*Bacteroidetes* ratio was thought to be related to many diseases such as obesity, type 2 diabetes, and chronic kidney disease, which was consistent with our study (Grigor’eva, 2020; Guan et al., 2020). We inferred that the immune disorder and renal damage caused by death of gut microbiome were more serious than the recovery of renal function caused by the reduction of uremic toxin producing bacteria.

Gut microbiome was widely applied to establish diagnostic models for various diseases thanks to its advantages of being noninvasive and safe (Ren et al., 2019; Yu et al., 2020; Ren et al., 2021; Shi et al., 2021). In our project, we also constructed a diagnostic model for MCD and validated it in different cohorts. Our results suggested that the model had good diagnostic capability and a wide application scope. Out of the optimum eight OTUs we obtained, OTU108 (*Solobacterium*), OTU92 (*Streptococcus*), OTU148 (*Pseudomonas*) and OTU64 (*Granulicatella*) were found to be positively correlated with SCr and negatively correlated with ALB, which may contribute to MCD progression and severity. *Solobacterium* primarily colonizes the mouth and is related to oral malodor (Vancauwenberghe et al., 2013) and bacteremia (Liu et al., 2019). However, we did not find any related studies in other systems. Most species of *Streptococcus* are pathogenic bacteria or opportunistic pathogens such as *Streptococcus pneumoniae* and *Streptococcus haemolyticus. Pseudomonas* was also recognized as a pathogenic bacterial strain, especially tending to cause severe pneumonia. A case report revealed chronic catheter-related bacteremia of *Pseudomonas stutzeri* etiology as the cause of membranous-proliferative glomerulonephritis (MPGN) (Cołoś et al., 2021). *Granulicatella* is considered a strain of pathogenic bacteria that causes infective endocarditis. It has been reported that *Granulicatella* infection could induce endocarditis and glomerulonephritis (Shaik et al., 2020). The increase in these pathogens might be one of the reasons for the aggravation of MCD.

In our study, we used DOX to induce MCD rat model. In the altered gut microbiome, *Oscillibacter* was a uremic metabolite producing bacteria and was found more abundant in patients with chronic kidney disease (Chen et al., 2022). *Alistipes* was another opportunistic pathogen, leading inflammation and even tumor (Moschen et al., 2016). Accumulation of these harmful bacteria might aggravate kidney damage in MCD. The increased abundance of *Coprococcus*, members of which were SCFA producing bacteria, seemed different from the assumption. We speculated that it might be related to the change of gut environment and partial compensatory increase. However, as anthracycline antibiotic, DOX might affect the abundance of gut microbiota. In some other animal models induced by DOX, the decrease of intestinal flora abundance was also observed (Bawaneh et al., 2022; Huang et al., 2022). However, we found that MCD rat model are still established successfully after gut microbial cleaning, which indicated that DOX did not induce MCD through gut microbiota. Gut microbiota are thought closely related to the health of the host (Rooks and Garrett, 2016; Lin and Zhang, 2017). Therefore, more and more animal experiments showed that gut microbiome interventions can treat or delay the disease (Bermudez-Martin et al., 2021; Rao et al., 2021). Clinical trials found FMT to be effective for diarrhea-predominant irritable bowel syndrome (Singh et al., 2022). Recently, FMT has also been used to treat membranous nephropathy as mentioned in a case report (Zhou et al., 2021). We also found that, under medication, gut microbiota compositions in MCDs were influenced to transform into those in healthy individuals. An analysis of the gut microbial composition showed that FMT was performed successfully in our project, while it seemed to have no treatment effect on MCD in animal experiments. We inferred that the onset of MCD caused gut dysbiosis, it means the alteration of gut microbiota likely occurred after the onset of MCD and it is difficult for FMT to eradicate the causes of MCD. Thus, treatment to the disease could partly recover the gut dysbiosis in MCD patients. However, more evidence is needed to confirm the therapeutic efficiency of FMT.

There are several innovations and advantages in this project. First of all, it was a large-sampled and multi-centered study involving 222 fecal samples from two medical centers. Secondly, we developed and validated a discriminative model for MCD diagnosis, and this diagnostic model provided a non-invasive and broadly-applied tool for clinicians. Our study had some limitations. The mechanism of gut microbial changes in MCD patients has not been investigated clearly. In addition, gut microbiota compositions in humans and rats are quite different. The dysbiosis with MCD patients and that induced with doxorubicin rat model is difficult to compare. The effect of FMT interventions on gut microbiota also needs to be further validated clinically.

## 5 Conclusion

The gut microbiota composition of MCD patients changed sharply in both humans and rats. Specific OTUs could be used as biomarkers to diagnose MCD. The discriminative model, with its high diagnostic efficiency, was suitable for MCD patients in different states. In animal experiments, FMT intervention did not have significant effects on MCD.

## Data Availability

The datasets presented in this study can be found in online repositories. The names of the repository/repositories and accession number(s) can be found below: https://www.ebi.ac.uk/ena, PRJNA752445 https://www.ebi.ac.uk/ena, PRJNA832071.
