# Supplementary figures and images for: Characterization and diagnostic value of the gut microbial composition in patients with minimal change disease
Source: Front Physiol. 2022 Dec 6;13:1070569. doi: 10.3389/fphys.2022.1070569 (PMC9763583; doi:10.3389/fphys.2022.1070569)

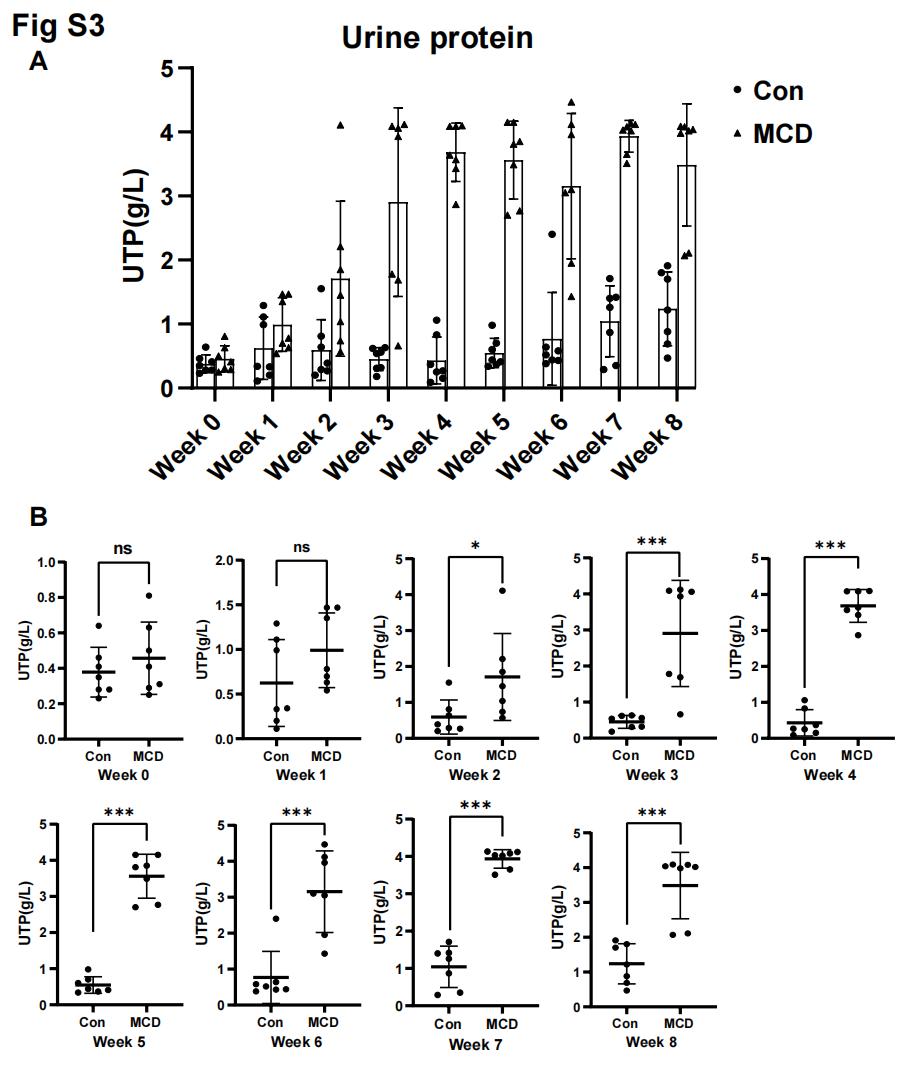

Supplement: Supplementary file 3 [file Image3.JPEG]

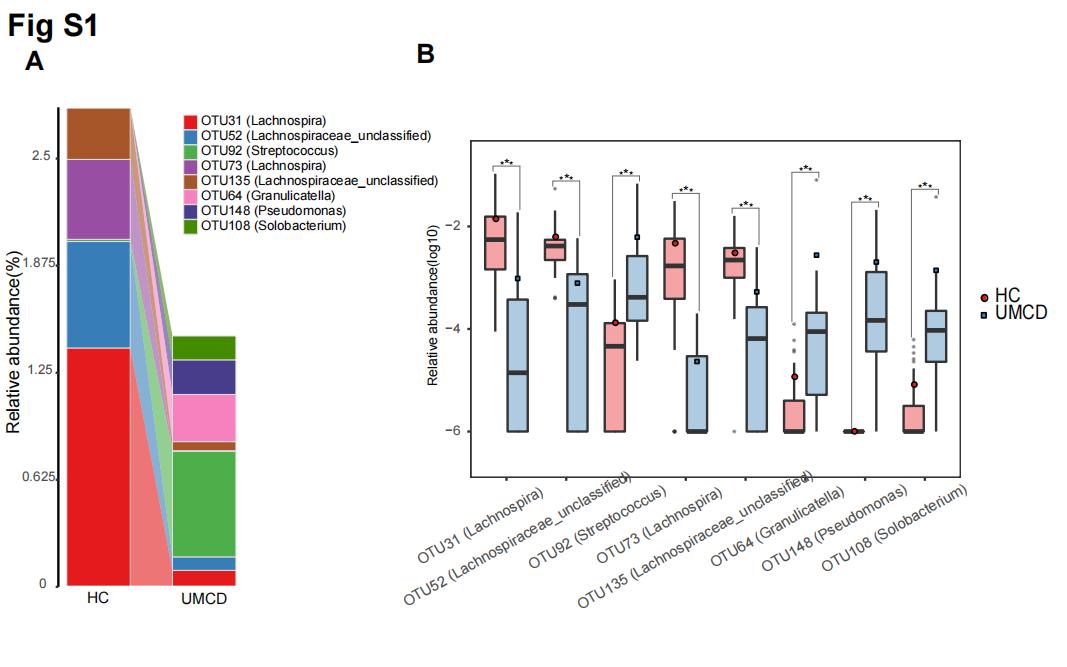

Supplement: Supplementary file 6 [file Image1.JPEG]

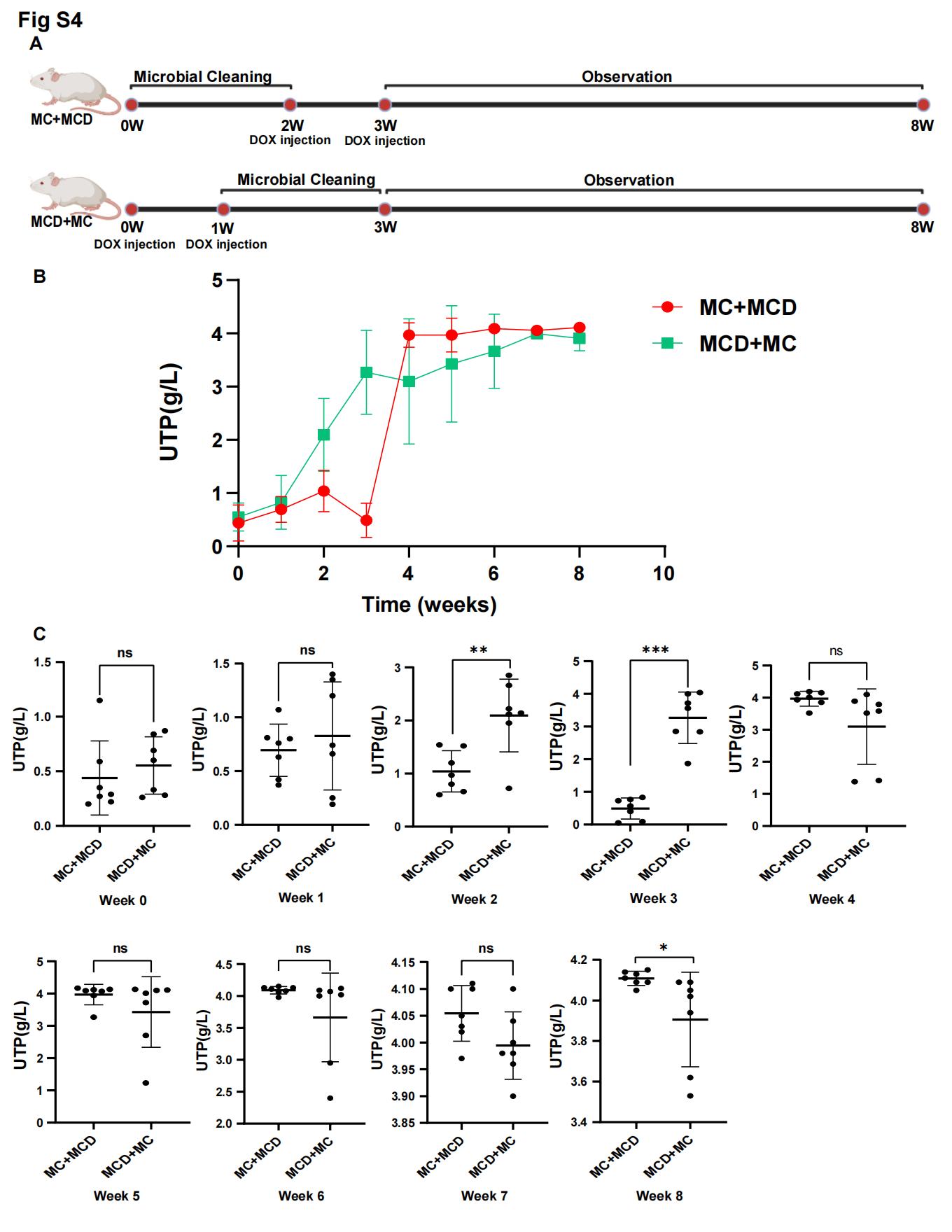

Supplement: Supplementary file 7 [file Image4.JPEG]

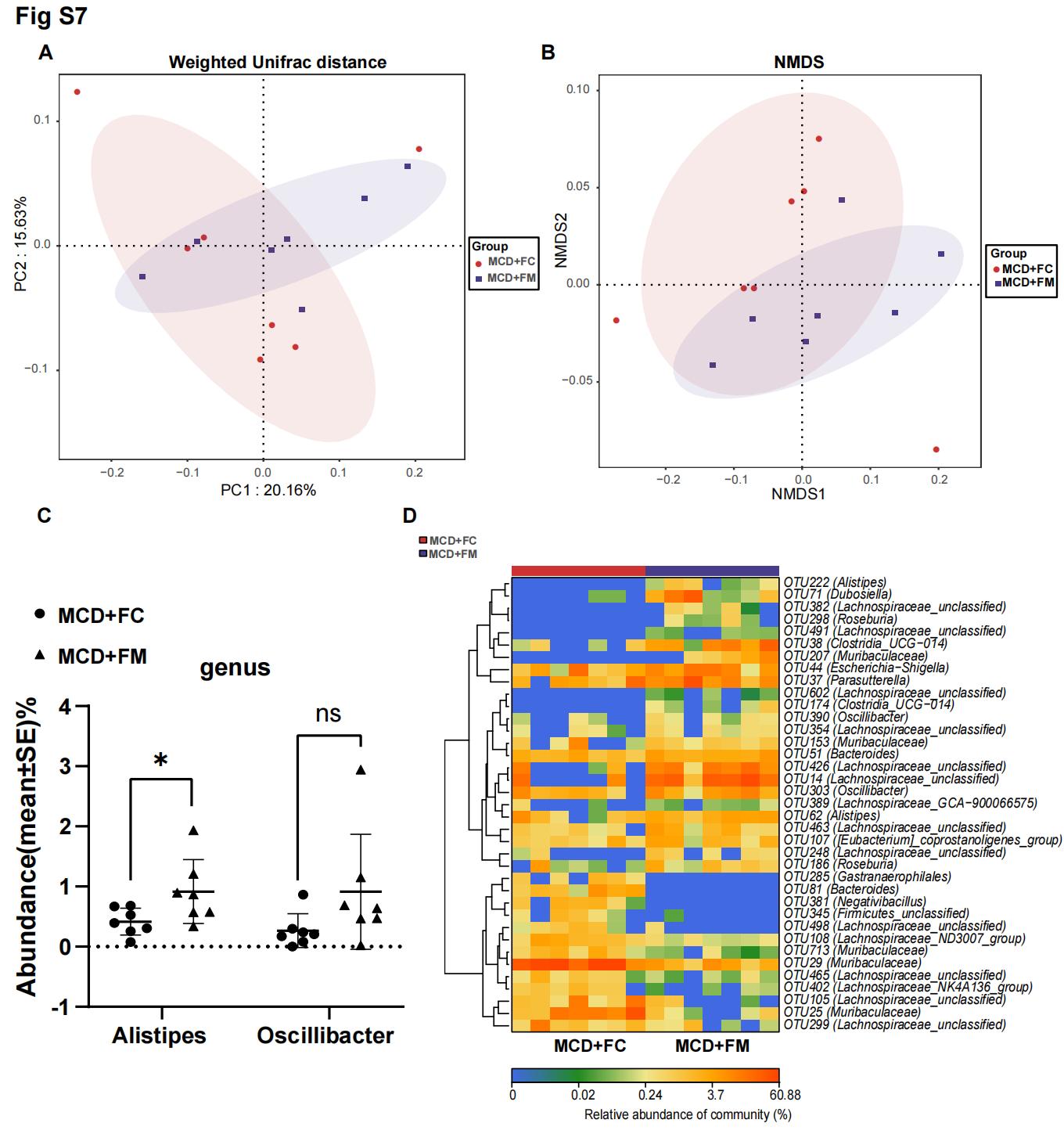

Supplement: Supplementary file 9 [file Image7.JPEG]

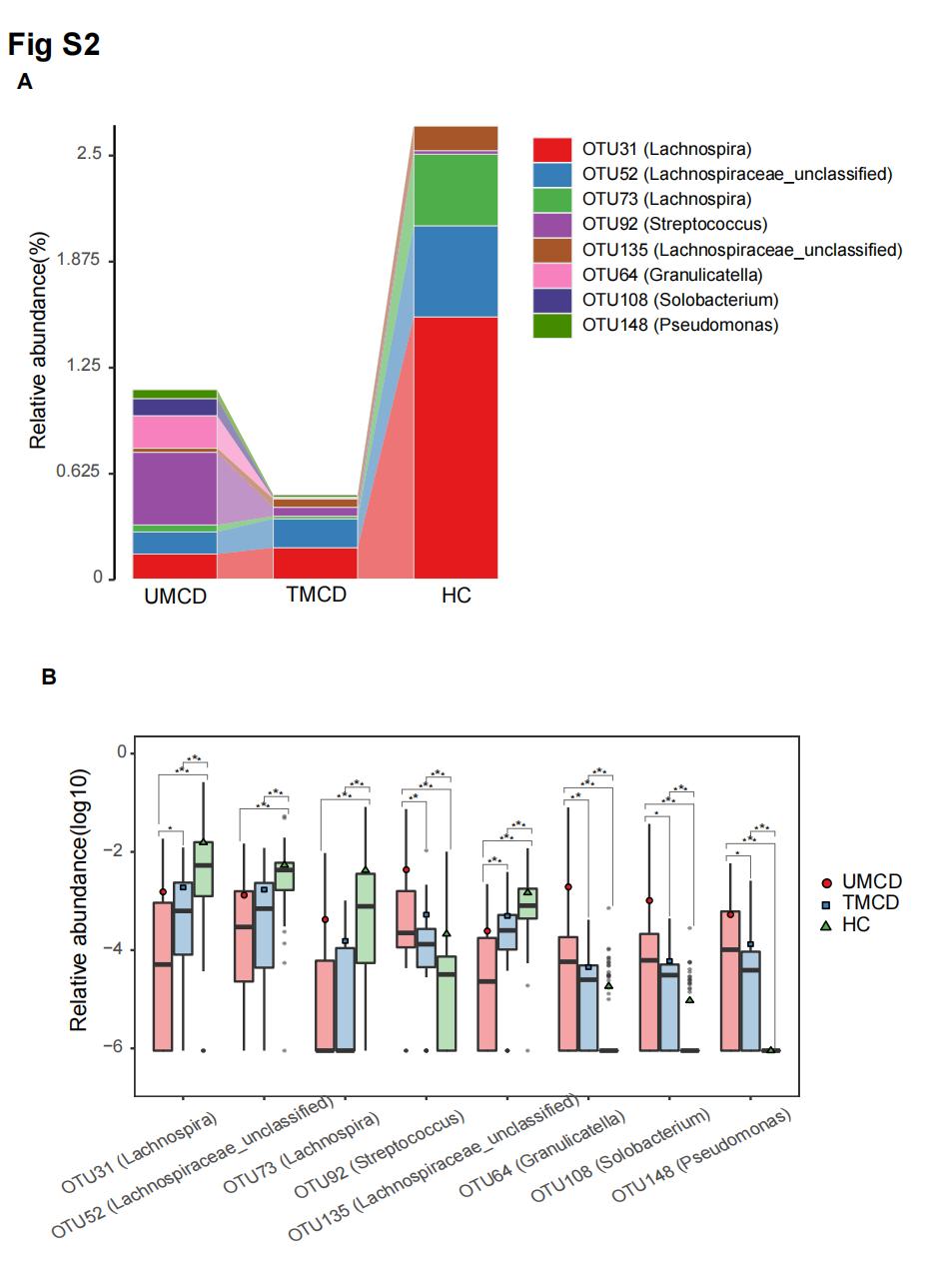

Supplement: Supplementary file 10 [file Image2.JPEG]

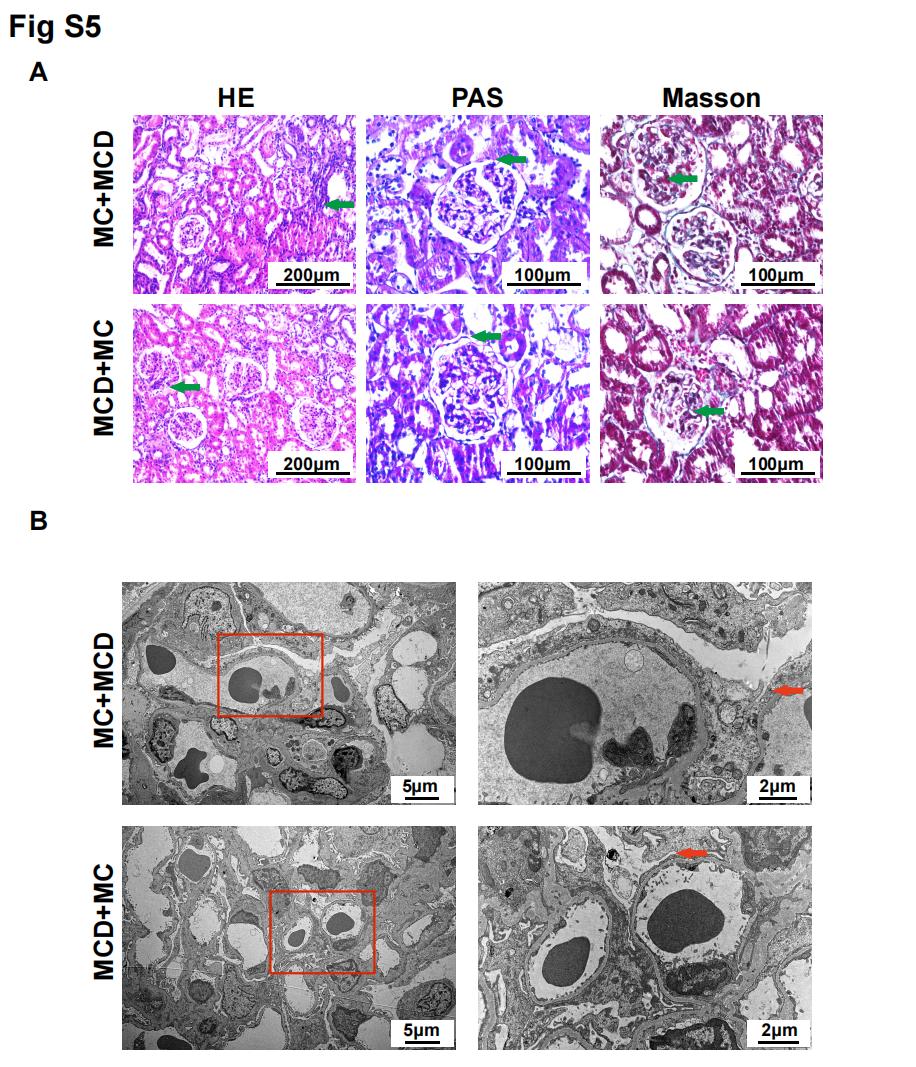

Supplement: Supplementary file 11 [file Image5.JPEG]

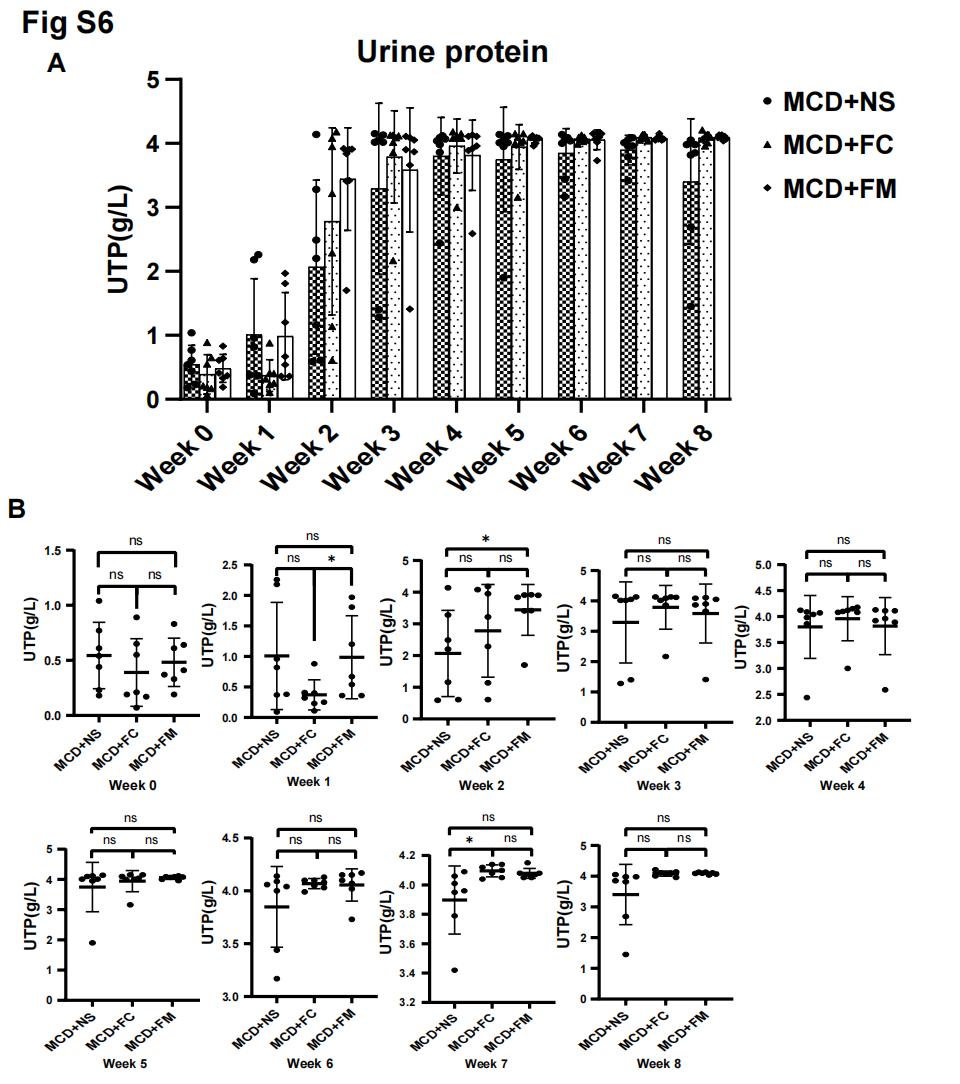

Supplement: Supplementary file 23 [file Image6.JPEG]
